# Biomarkers Characterizing the Onset of Dietary-Induced Hepatocellular Injury and Visceral Obesity in a Rat Experimental Model: Possible Anti-Inflammatory Effects of Steviol Glycosides

**DOI:** 10.3390/metabo15100656

**Published:** 2025-10-04

**Authors:** Krastina Trifonova, Penka Yonkova, Petko Dzhelebov

**Affiliations:** 1 Department of General and Clinical Pathology, Faculty of Veterinary Medicine, Trakia University, Stara Zagora 6015, Bulgaria; krastina.trifonova@trakia-uni.bg; 2 Department of Veterinary Anatomy, Histology and Embryology, Faculty of Veterinary Medicine, Trakia University, Stara Zagora 6015, Bulgaria; penka.yonkova@trakia-uni.bg

**Keywords:** high-carbohydrate diet, high-fat diet, liver injury, rodent model, steviol glycosides, visceral obesity

## Abstract

**Background:** The aim of the present study is to compare the potential of a high-fat diet, a high-carbohydrate diet, and a high-fat, high-carbohydrate diet to induce liver injury and visceral obesity within a period of five weeks, identify the pattern and degree of hepatic changes at the tissue level, identify the earliest metabolic markers of specific liver changes induced by each type of diet, and to test the possible beneficial effects of steviol glycosides in a rat experimental model. **Methods:** Wistar rats (n = 56) were divided into seven groups as follows: group BD (before diet), group SD (standard diet), group HFD (high-fat diet), group HCHD (high-carbohydrate diet), group HFHCHD (high-fat high-carbohydrate diet), group SDS (standard diet supplemented with Stevia extract), and group HFDS (high-fat diet supplemented with Stevia extract). **Results:** Total cholesterol concentrations (2.02 ± 0.22 mmol/L) increased in the HFD group (2.56 ± 0.82 mmol/L) and in the HFDS group (2.89 ± 0.48 mmol/L). The VLDL values before diets were 0.27 ± 0.11 mmol/L and increased most significantly in the HFHCHD group—1.14 ± 0.62 mmol/L. The baseline ALT values (88.4 ± 10.6 U/L) increased in the HFD group (128.13 ± 19.5 U/L) and the HFDS group (127.00 ± 17.74 U/L). Similar increases were registered in the AST/ALT ratio and ALP. Total bilirubin (7.10 ± 1.39 μmol/L) increased in HFD group (27.86 ± 17.01 μmol/L). Serum NO had the lowest values in groups fed diets supplemented with steviol glycosides. All high-calorie diets induced hepatocellular injury. The mass of the perirenal fat depot and cross-sectional area of adipocytes were highest in HFD, HFHCHD, and HFDS groups. **Conclusion:** High-calorie diets have the potential to induce visceral obesity and hepatocellular injury within a very short period of time, which produces characteristic histological changes and specific biochemical profile. Steviol glycosides may alleviate some aspects of the inflammatory response, but findings about lipid profile parameters and liver enzymes are controversial.

## 1. Introduction

The liver plays a central role in the body, regulating various functions—it regulates the metabolism of macronutrients, it is also responsible for the metabolism of some hormones and the biotransformation of many chemicals, including medications, it is involved in the production of clotting factors, the synthesis and storage of vitamins, and several others [1].

Various hepatic injuries may render the liver unable to regulate lipid, protein, and carbohydrate metabolism, and in some cases this results in hepatic insulin resistance [2]. In general, insulin resistance is defined as defective insulin-stimulated glucose uptake into skeletal muscle cells and adipocytes. Usually in addition to this, insulin suppression of hepatic glucose output is also being impaired [3,4]. Thus, very often liver injuries are characterized by hyperglycemia, which could become a subsequent trigger for the onset of diabetes type 2 [5,6]. Because of the abovementioned, it has been concluded that there might be a significant and complex association between some liver injuries and diabetes type 2. Having in mind the epidemic prevalence of diabetes, scientific research related to liver disorders recently has become extremely important. Various causes of liver injuries have been identified—alcohol consumption, toxins, infections, medications, dietary supplements, and nutrition [7]. In the context of diabetes type 2, non-alcoholic fatty liver disease, and several other obesity-related diseases, nutritional excess seems to play an important role.

In general, there are four patterns of liver injury—hepatocellular, autoimmune, cholestatic, and infiltrative—each being associated with inflammatory reaction. Abnormal values of liver tests are a common finding in many patients who have no clinical manifestation of liver disease [8]. Thus, identifying the biochemical profile of each pattern of liver injury is very important so that treatment measures can be taken before the liver disease becomes apparent and histological changes become irreversible. While most human studies usually focus on late consequences of liver disease, experimental research using animal models of liver injury offers the advantage of studying the onset of disorders, their progression, and their advanced stages. Various animal models of liver injuries have been described in the scientific literature. Such liver injury models are useful to investigate the mechanisms of liver disease, to identify potential diagnostic and prognostic biomarkers, to test hepatoprotective drugs, and to set principles of prevention [9,10]. For these reasons, nowadays the development of standard animal models has become the basis of disease research. Various rodent models of chemical liver injury have been established—such models use carbon tetrachloride, α-naphthyl isothiocyanate, diethylnitrosamine, thioacetamide, and others. In addition to this, immunological and drug-induced rodent models of liver injury are also being used [5]. The classical acute and chronic rodent models of alcohol-associated liver disease are also of great importance [11]. But in the context of obesity-related and metabolic diseases, the dietary rodent models seem to be of the greatest significance, as the disorders they induce can progress to non-alcoholic fatty liver disease or diabetes type 2 [12,13]. Three main experimental dietary regimens in rodents have been widely used in biomedical research related to obesity and metabolic diseases: high-fat diet (HFD), high-carbohydrate diet (HCHD), and high-fat high-carbohydrate diet (HFHCHD) [14]. These diets have been applied in various rodent species, with different durations, and with varying concentrations and types of the added fats and carbohydrates depending on the aim of the study. Most of the studies using these experimental diets focus on weight gain and some metabolic aspects and have reported the development of obesity with visceral fat accumulation, abnormal lipid profiles, and insulin resistance [15,16,17]. Just a few of the studies using these diets have investigated the onset of liver changes at the histological level.

As mentioned earlier, animal experimental models are also useful for the development of new prevention and treatment methods. In recent decades the scientific literature abounds with plenty of studies on the therapeutic potential of various plant extracts, nutritional supplements, and herbs [18]. Stevia extract, which is derived from the sweet plant *Stevia Rebaudiana*, recently has become extremely popular as a sweetener. In addition to this, research on the intake of steviol glycosides shows that they may have anti-inflammatory and anti-diabetic effects [19,20].

Thus, the aim of the present study is to compare the potential of a high-fat diet, a high-carbohydrate diet, and a high-fat, high-carbohydrate diet to induce liver injury and visceral obesity within a short period of time, identify the pattern and degree of hepatic changes at the tissue level, identify earliest metabolic markers of specific liver changes induced by each type of diet, and to test the possible anti-inflammatory effects of steviol glycosides in a rat experimental model.

## 2. Materials and Methods

Experimental animals

The research was approved by the Animal Ethics Committee of the Bulgarian Food Safety Agency (Permit № 335; with opinion of the Ethics Committee № 251 of 19.10.2022).

A total of 56 male Wistar rats, at ~9 weeks of age, with an initial body weight of 160 ± 20 g, were used in the study. Animals were purchased from the breeding facility of Medical University, Plovdiv, Bulgaria. The experimental animals were kept in cages indoors. Controlled environmental conditions (room temperature of 22 ± 2 °C, 40–70% relative humidity, 12:12 h light-dark cycle) were provided. The adaptation period lasted two weeks. All animals had free access to feed and water throughout the entire experimental period. In relation to the goals of the experiment, the diet of some of the formed groups was manipulated.

Experimental groups and diets

During the adaptation period, all animals were fed a standard pelleted feed for rodents (Melhran, LTD, Stara Zagora, Bulgaria). This standard feed had an energy content of 290 kcal/100 g (6.75% of calories provided by fat, 69.21% of calories provided by carbohydrates, and 24.04% of calories by protein). For the purpose of the study, rats were divided into 7 equal groups (n = 10). For some of the experimental groups, the diet was manipulated by means of adding to the standard diet a certain amount of lard, sucrose, Stevia extract*, or various combinations of these. The animals were submitted to the manipulated dietary regimens for a period of 5 weeks.

Group BD (before diet)—animals from this group were fed ad libitum the standard rodent feed (Melhran, LTD, Bulgaria). Group BD was used to measure the baseline values of the studied parameters before the start of the dietary regimens.

Group SD (standard diet)—rats from this group were fed ad libitum the same standard rodent feed for the entire experimental period (5 weeks).

Group HFD (high-fat diet)—animals from this group were fed the standard rodent feed supplemented with lard in such an amount that the energy provided by fat was 40% of the total energy intake.

Group HCHD (high-carbohydrate diet)—this group received the standard rodent feed supplemented with sucrose (75% of the energy content provided by carbohydrates).

Group HFHCHD (high-fat high-carbohydrate diet)—rats were fed the standard rodent feed, to which lard and sucrose were added in such amounts that the energy derived from fats was 30% and energy from carbohydrates was 57%.

Group SDS (standard diet plus Stevia extract)—rats were fed the standard pelleted feed supplemented with Stevia extract (Balgarska Stevia, LTD, Veliko Tarnovo, Bulgaria); this diet was used to evaluate possible positive effects of decreasing the energy content of the HCHD by substituting sucrose with stevia extract according to the instructions of the manufacturer.

Group HFDS (high-fat diet plus Stevia extract)—rats were fed the standard pelleted feed enriched in lard (same amount as in high-fat high-carbohydrate diet) and Stevia extract (replacing the sucrose in the high-fat, high-carbohydrate diet); this diet was used to test potential benefits of reducing the calorie content of the HFHCHD by substituting sucrose with Stevia extract.

*Contents of Stevia extract* (Balgarska Stevia, LTD, Bulgaria): 96.8% of steviol glycosides as follows—61.06% rebaudioside A, 30.36% stevioside, 12.42% rebaudioside C, 2.49% dulcoside A, 0.2% steviolbioside, 0.16% rebaudioside B, 0.12% rebaudioside. The certificate for chemical analysis of the extract was provided by the manufacturer.

Samples

Blood samples were taken at the end of the 5-week dietary regimen for each group. Blood samples were collected after overnight fasting by retro-orbital bleeding under general anesthesia: Xylazine—8 mg/kg + Ketamine—90 mg/kg; i.p. (Xylazine, Alfasan International BV, Woerden, The Netherlands; Anaket, Richter Pharma AG, Wels, Austria).

To separate serum, blood samples were collected in tubes without anticoagulant and kept at room temperature. After complete clotting, the tubes were centrifuged for 10 min at RCF 1500× *g* (Centurion 2040, Centurion Scientific Ltd., Chichester, UK).

To obtain plasma, blood was collected in heparin tubes (Lind-Vac^®^, Intervactechnology OÜ, Narva, Estonia) and was centrifuged for 15 min at RCF 1500× *g*.

To perform light microscopic examinations, liver tissue and perirenal fat tissue were taken—six rats from each group were euthanized by cervical dislocation after induction of xylazine-ketamine anesthesia. Liver tissue samples were obtained from the right medial liver lobe. Respectively, the right and left parts of the perirenal fat depot were removed. The mass of the perirenal adipose depot (in grams, g) was measured using a digital analytical balance AQT-200 (ADAM Equipment Inc., Danbury, CT, USA).

The hepatic and fat tissue samples were fixed for 48 h in a 10% neutral buffered formalin solution (Diapath S.p.A., via Pietro Savoldini, 71, 24057 Martinengo (BG), Italy; CAS: NA), then replaced into an embedding cassette (Ref 0106-1100-12, CITOTEST LABWARE MANUFACTURING CO., Ltd., Halmen City, China). The following steps of washing, dehydration of the tissues in a series of increasing alcohol concentrations, clearing in xylene, and infusion with molten paraffin were performed in a tissue processor, HistoCore PEARL Tissue Processor (Leica Biosystems, Switzerland AG). Paraffin infiltrated hepatic and fat tissues were sliced with a thickness of 3-5 μm on a rotary microtome Leica RM 2235 (Leica Microsystems, Nussloch, Germany). The slices were stained using standard hematoxylin-eosin methodology.

Light microscopic examinations on the liver parenchyma were aimed at qualitatively assessing tissue integrity, vascular changes, and the presence of pathological infiltrates. The assessment of perirenal adipose tissue was based on the change in the size of adipocytes. In selected regions, in which adipocytes had preserved cell membrane integrity and a clearly visible nucleus, the cross-sectional area (in μm^2^) of 50 adipocytes was measured using Leica Application Suite (LAS, version 4.8.0., Leica Microsystems CMS, GmBH Heerbrugg, Switzerland).

Biochemical parameters—lipid profile parameters (TC, HDL, VLDL, TG), enzymes (AST, ALT, ALP), and total bilirubin were measured using a biochemical analyzer, Mindray BS-120 (Mindray Medical International Limited). The AST/ALT ratio was calculated.

Nitric oxide (NO-μmol/L)—concentrations of nitric oxide in serum samples were measured by the spectrophotometric method of Miranda [21]; the method was developed to measure NO concentrations in biological fluids.

Statistical analysis

Results data are presented as mean values ± standard deviation (SD). Statistical analysis is based on one-way analysis of variance (one-way ANOVA) followed by Tukey’s post hoc test; Graph Pad InStat 3.1 software was used. The level of statistically significant differences was set at *p* < 0.05.

## 3. Results

### 3.1. Lipid Profile Parameters

**Figure 1:**
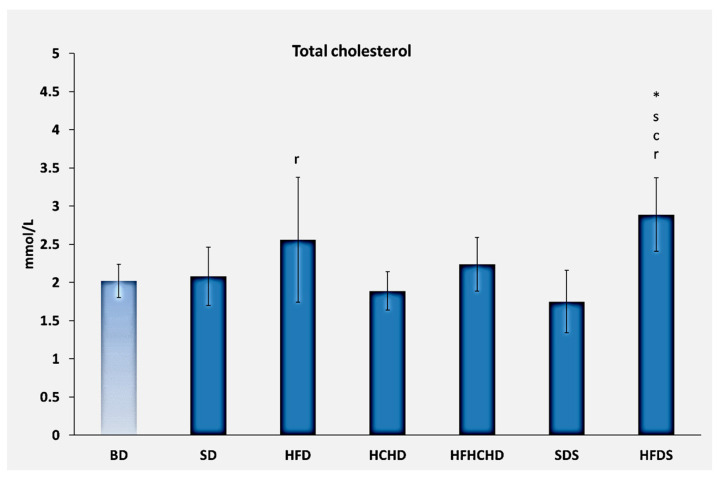
Total blood cholesterol concentrations in rats (n = 8) before the start of the diets (BD) and in groups SD (n = 8), HFD (n = 8), HCHD (n = 8), HFHCHD (n = 8), SDS (n = 8), and HFDS (n = 8) after 5 weeks of application of the respective diets. Results are presented as mean values ± standard deviation. Statistically significant differences are indicated as follows: * *p* < 0.05 vs. BD; s *p* < 0.05 vs. SD; c *p* < 0.05 vs. HCHD; r *p* < 0.05 vs. SDS.

Total cholesterol concentrations before the start of the diets were 2.02 ± 0.22 mmol/L. After 5 weeks of application of the respective diets, total cholesterol increased statistically significantly in the HFD group (2.56 ± 0.82 mmol/L) compared to the SDS group. A significant increase was also recorded in the HFDS group (2.89 ± 0.48 mmol/L) compared to the BD, SD, HCHD, and SDS (*p* < 0.05) (Figure 1).

**Figure 2:**
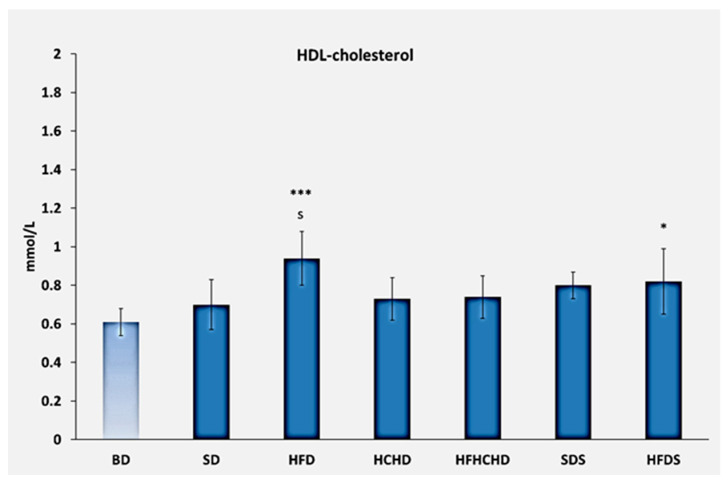
Concentrations of HDL-cholesterol in the blood of rats (n = 8) before the start of the diets (BD) and in groups SD (n = 8), HFD (n = 8), HCHD (n = 8), HFHCHD (n = 8), SDS (n = 8), and HFDS (n = 8) after 5 weeks of application of the respective diets. The results are presented as mean values ± standard deviation. Statistically significant differences are indicated as follows: * *p* < 0.05 vs. BD; *** *p* < 0.001 vs. BD; s *p* < 0.05 vs. SD.

The baseline values of HDL-cholesterol were 0.61 ± 0.07 mmol/L. After applying the respective diets for a period of 5 weeks, values increased in the HFD group compared to the BD group (*p* < 0.001) and compared to the SD group (*p* < 0.05). A significant increase was also registered in the HFDS group compared to the BD group (*p* < 0.05) (Figure 2).

**Figure 3:**
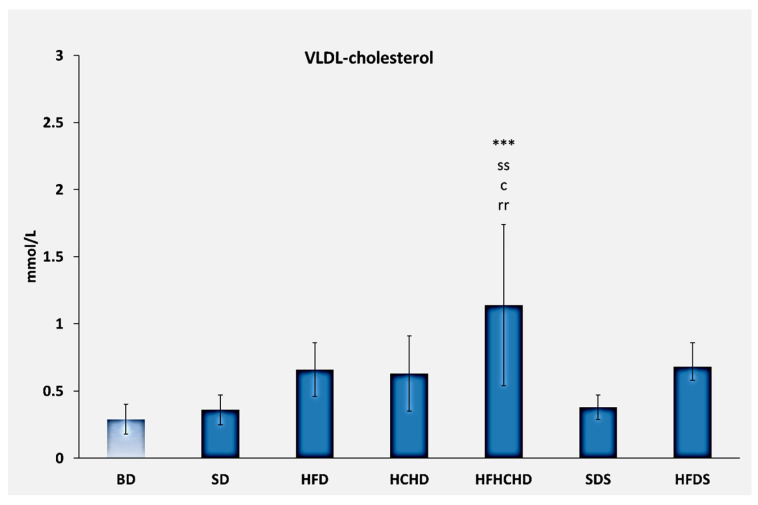
Concentrations of VLDL-cholesterol in the blood of rats (n = 8) before the start of the diets (BD) and in groups SD (n = 8), HFD (n = 8), HCHD (n = 8), HFHCHD (n = 8), SDS (n = 8), and HFDS (n = 8) after 5 weeks of application of the respective diets. The results are presented as mean values ± standard deviation. Statistically significant differences are indicated as follows: *** *p* < 0.001 vs. BD; ss *p* < 0.01 vs. SD; c *p* < 0.05 vs. HCHD; rr *p* < 0.01 vs. SDS.

The VLDL-cholesterol values recorded before the start of the diets were 0.27 ± 0.11 mmol/L. After 5 weeks of application of the respective diets, values increased in the HFD, HCHD, HFHCHD, and HFDS groups. The highest values were measured in the HFHCHD group—1.14 ± 0.62 mmol/L, and they were significantly higher compared to BD (*p* < 0.001), SD (*p* < 0.01), HCHD (*p* < 0.05), and SDS (*p* < 0.01) (Figure 3).

**Figure 4:**
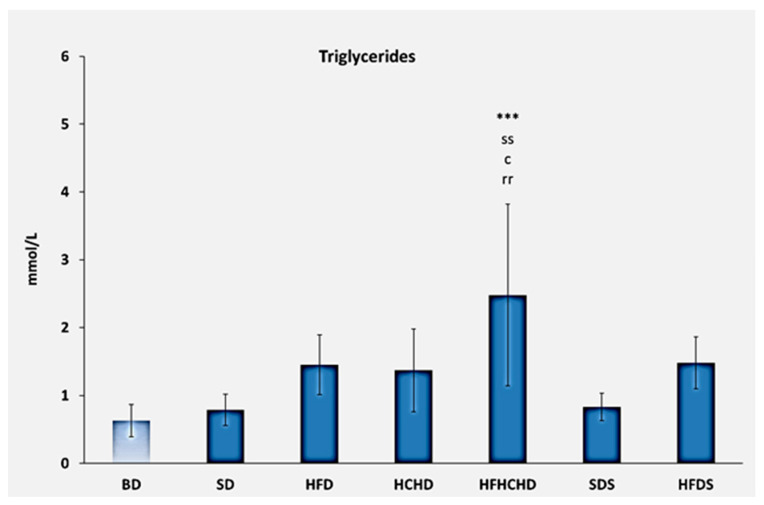
Blood triglyceride concentrations in rats (n = 8) before the start of the diets (BD) and in the groups SD (n = 8), HFD (n = 8), HCHD (n = 8), HFHCHD (n = 8), SDS (n = 8), and HFDS (n = 8) after 5 weeks of application of the respective diets. The results are presented as mean values ± standard deviation. Statistically significant differences are indicated as follows: *** *p* < 0.001 vs. BD; ss *p* < 0.01 vs. SD; c *p* < 0.05 vs. HCHD; rr *p* < 0.01 vs. SDS.

Triglyceride levels before the start of the diets were 0.63 ± 0.24 mmol/L. After 5 weeks of application of the respective diets, increased values were recorded in the HFD, HCHD, HFHCHD, and HFDS groups. The highest values were measured in the HFHCHD group—2.48 ± 1.34 mmol/L—and they were significantly higher compared to BD (*p* < 0.001), SD (*p* < 0.01), HCHD (*p* < 0.05), and SDS (*p* < 0.01) (Figure 4).

### 3.2. Liver Enzymes

**Figure 5:**
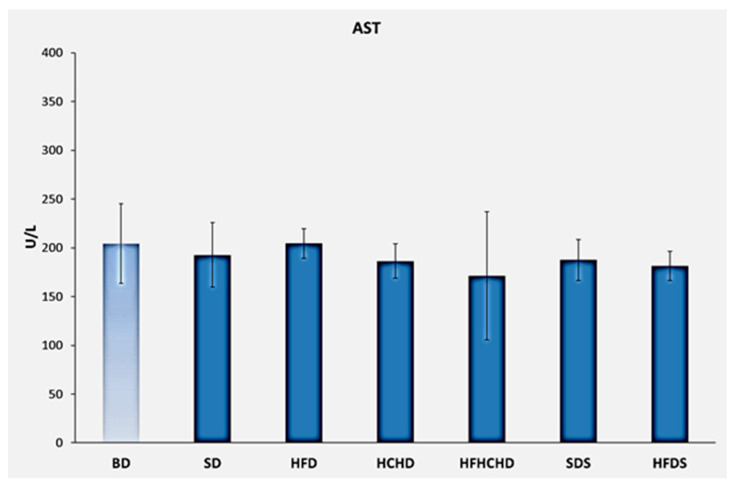
Blood AST concentrations in rats (n = 8) before the start of the diets (BD) and in groups SD (n = 8), HFD (n = 8), HCHD (n = 8), HFHCHD (n = 8), SDS (n = 8), and HFDS (n = 8) after 5 weeks of application of the respective diets. Results are presented as mean values ± standard deviation.

The baseline AST values were 204.29 ± 40.76 U/L. After applying the respective diets for a period of 5 weeks, no statistically significant changes were found either compared to the baseline level or between the different groups (Figure 5).

**Figure 6:**
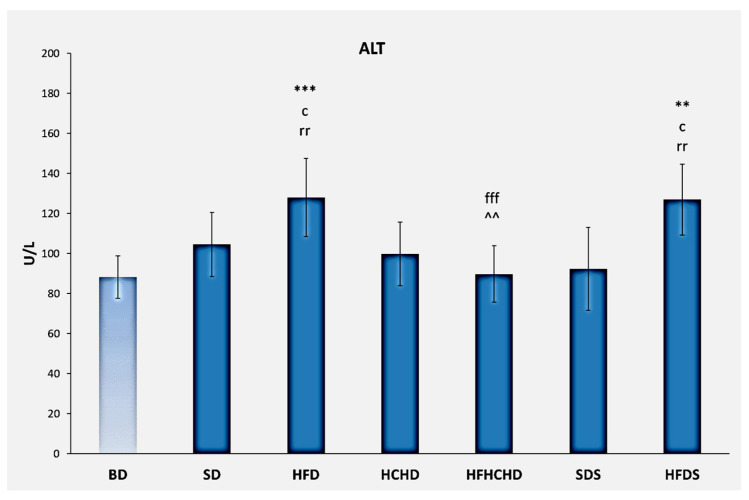
Blood ALT concentrations in rats (n = 8) before the start of the diets (BD) and in groups SD (n = 8), HFD (n = 8), HCHD (n = 8), HFHCHD (n = 8), SDS (n = 8), and HFDS (n = 8) after 5 weeks of application of the respective diets. Results are presented as mean values ± standard deviation. Statistically significant differences are indicated as follows: *** *p* < 0.001 vs. BD; ** *p* < 0.01 vs. BD; c *p* < 0.05 vs. HCHD; rr *p* < 0.01 vs. SDS; fff *p* < 0.001 vs. HFD; ^^ *p* < 0.01 vs. HFDS.

The baseline ALT values were 88.42 ± 10.61 U/L. After applying the respective diets for 5 weeks, the values increased in animals from the HFD group (128.13 ± 19.51 U/L). The increase was significant compared to BD (*p* < 0.001), HCHD (*p* < 0.05), SDS (*p* < 0.01), and HFHCHD groups (*p* < 0.001). A similar increase in the values of this parameter was also recorded in the animals of the HFDS group. This increase was significant compared to BD (*p* < 0.01), HCHD (*p* < 0.05), HFHCHD, and SDS (*p* < 0.01) (Figure 6).

**Figure 7:**
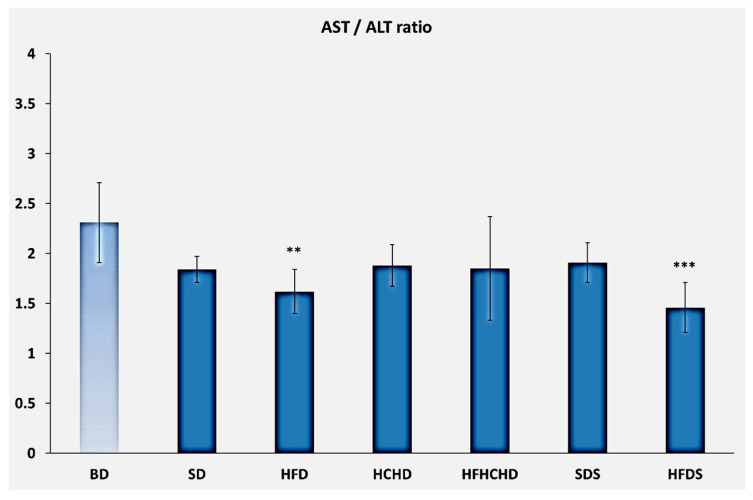
AST / ALT ratio in rats (n = 8) before the start of the diets (BD) and in groups SD (n = 8), HFD (n = 8), HCHD (n = 8), HFHCHD (n = 8), SDS (n = 8), and HFDS (n = 8) after 5 weeks of application of the respective diets. Results are presented as mean values ± standard deviation. Statistically significant differences are indicated as follows: *** *p* < 0.001 vs. BD; ** *p* < 0.01 vs. BD.

The initial levels of the AST/ALT ratio were 2.31 ± 0.40. After applying the respective diets for a period of 5 weeks, the values decreased in all groups, but the decrease was significant only in HFD (*p* < 0.01) and HFDS (*p* < 0.001). In the HFD group the ratio was 1.62 ± 0.22, and in HFDS—1.46±0.25 (Figure 7).

**Figure 8:**
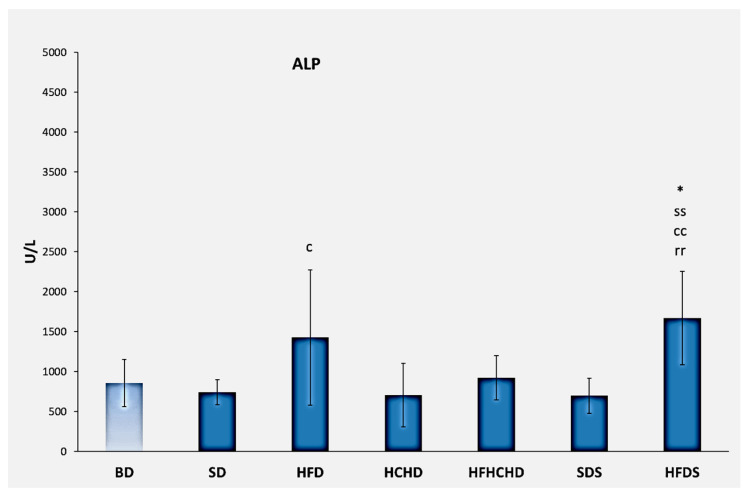
Blood alkaline phosphatase concentrations in rats (n = 8) before the start of the diets (BD) and in groups SD (n = 8), HFD (n = 8), HCHD (n = 8), HFHCHD (n = 8), SDS (n = 8), and HFDS (n = 8) after 5 weeks of application of the respective diets. Results are presented as mean values ± standard deviation. Statistically significant differences are indicated as follows: * *p* < 0.05 vs. BD; ss *p* < 0.01 vs. SD; c *p* < 0.05 vs. HCHD; cc *p* < 0.01 vs. HCHD; rr *p* < 0.01 vs. SDS.

The baseline values of alkaline phosphatase were 859.14 ± 295.59 U/L. After applying the respective diets for a period of 5 weeks, the values increased significantly in the HFDS group compared to the BD (*p* < 0.05) and SD, HCHD, and SDS groups (*p* < 0.01). The values of alkaline phosphatase also increased in rats from the HFD group, and the increase was significant compared to the HCHD group (*p* < 0.05) (Figure 8).

### 3.3. Total Bilirubin

**Figure 9:**
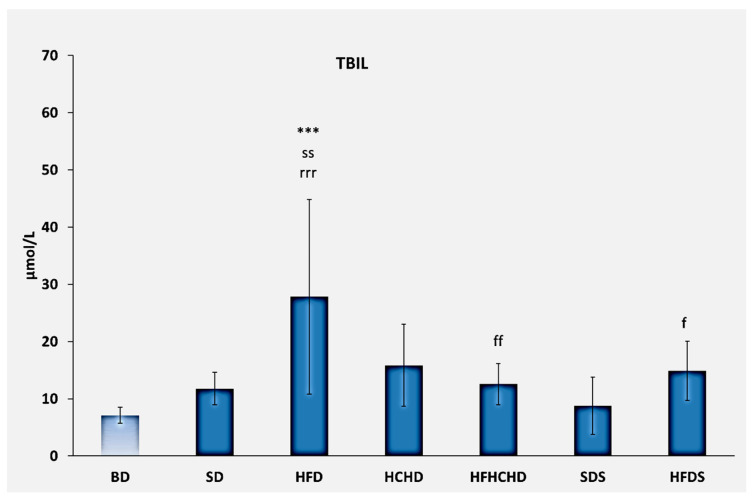
Total bilirubin concentrations in the blood of rats (n = 8) before the start of the diets (BD) and in groups SD (n = 8), HFD (n = 8), HCHD (n = 8), HFHCHD (n = 8), SDS (n = 8), and HFDS (n = 8) after 5 weeks of application of the respective diets. Results are presented as mean values ± standard deviation. Statistically significant differences are indicated as follows: *** *p* < 0.001 vs. BD; ss *p* < 0.01 vs. SD; f *p* < 0.05 vs. HFD; ff *p* < 0.01 vs. HFD; rrr *p* < 0.001 vs. SDS.

The baseline levels of total bilirubin were 7.10 ± 1.39 μmol/L. After 5 weeks on the respective diets, total bilirubin increased statistically significantly in rats from the HFD group compared to BD (*p* < 0.001), SD (*p* < 0.01), SDS (*p* < 0.001), HFHCHD (*p* < 0.01), and HFDS (*p* < 0.05) (Figure 9).

### 3.4. Nitric Oxide

**Figure 10:**
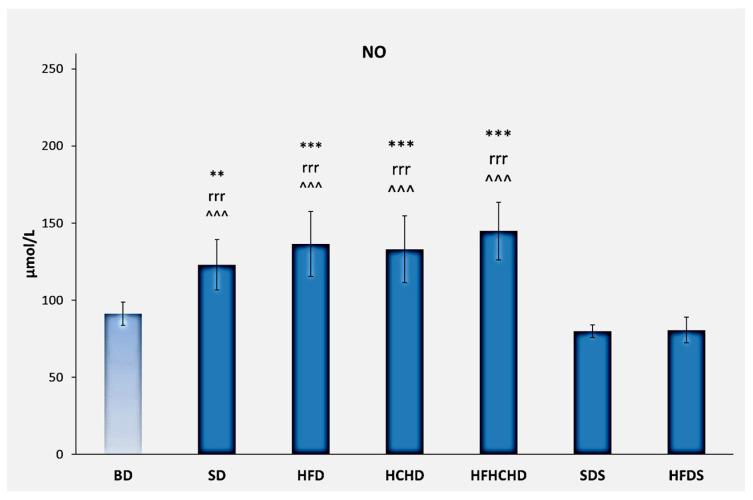
Nitric oxide concentrations in rats (n = 8) before the start of the diets (BD) and in groups SD (n = 8), HFD (n = 8), HCHD (n = 8), HFHCHD (n = 8), SDS (n = 8), and HFDS (n = 8) after 5 weeks of application of the respective diets. Results are presented as mean values ± standard deviation. Statistically significant differences are indicated as follows: ** *p* < 0.01 vs. BD; *** *p* < 0.001 vs. BD; rrr *p* < 0.001 vs. SDS; ^^^ *p* < 0.001 vs. HFDS.

The baseline NO concentrations were 91.25 ± 7.52 μmol/L. After applying the respective diets for a period of 5 weeks, nitric oxide increased in the SD, HFD, HCHD, and HFHCHD groups. The increase in SD was significant compared to BD (*p* < 0.01). Significant increases compared to BD were also registered in HFD, HCHD, and HFHCHD groups (*p* < 0.001). The increases in all groups were also statistically significant as compared to SDS and HFDS (*p* < 0.001) (Figure 10).

### 3.5. Liver Histology

**Figure 11:**
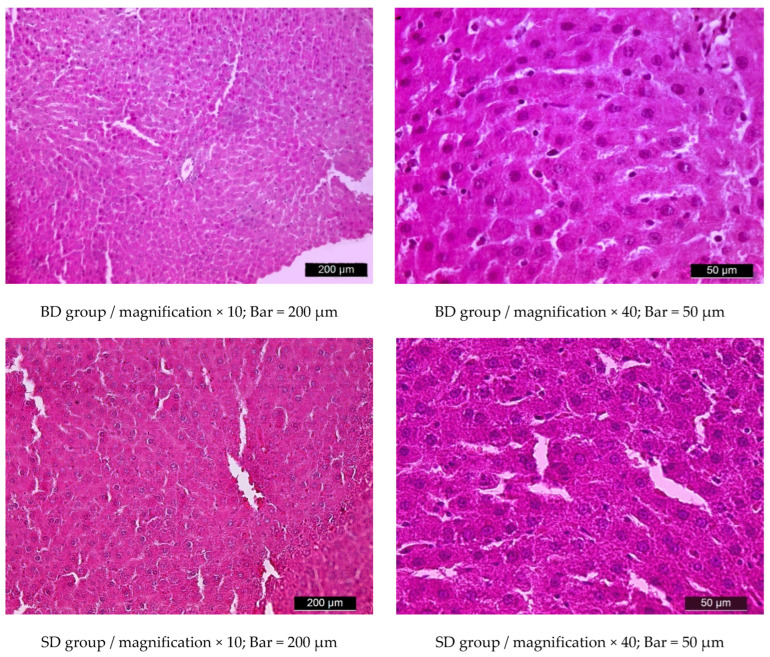
Light microscopic images of normal liver parenchyma in a rat from group BD and a rat from group SD. Hematoxylin–eosin staining.

Light microscopic observations revealed normal histological structure of the liver parenchyma in rats from groups BD and SD—normal structure of hepatic lobules and blood vessels, with the typical radial arrangement of hepatocyte plates (Figure 11).

**Figure 12:**
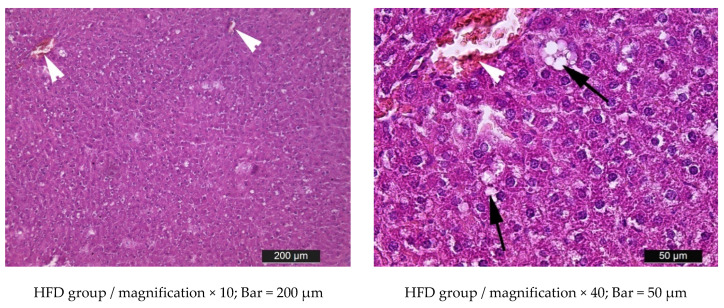

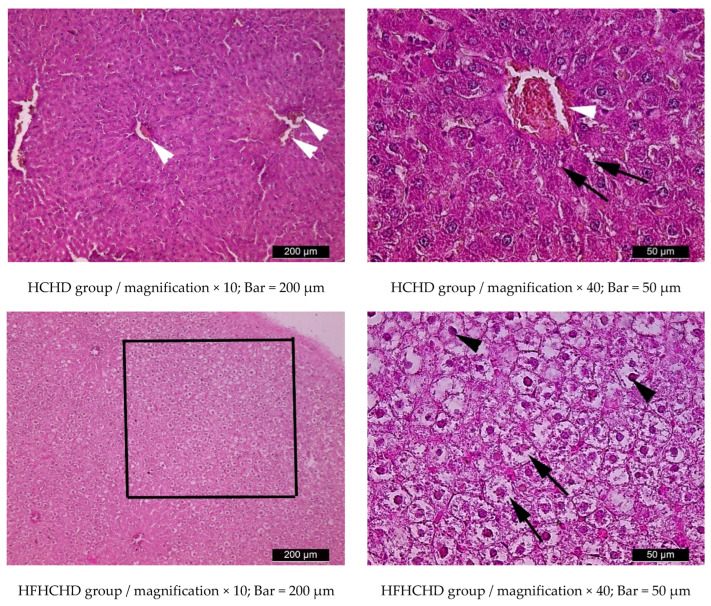
Light microscopic images of altered liver parenchyma in rats from HFD, HCHD, and HFHCHD groups. Black arrows indicate accumulation of lipids in the cytoplasm of hepatocytes (ranging from single fat droplets to aggregates of droplets (HFD)), single small fat droplets (HCHD), and confluent fat vacuoles (HFHCHD). White arrowheads—dilated central veins filled with blood; black arrowheads – hepatocytes with karyopyknosis; the square marks an area with impaired radial orientation of the liver plates. Hematoxylin–eosin staining.

In HFD group, areas with abnormal arrangement of the liver plates were found. Most of the hepatocytes were filled with intracytoplasmic lipid vacuoles. Moreover, most of the hepatocytes contained clearly visible multiple lipid vacuoles. This caused severe compression of the nuclei in the periphery of the liver cells, degeneration, and sometimes even complete destruction of hepatocytes. Some of the central and interlobular veins were dilated and filled with blood (Figure 12).

Rats from the HCHD group did not have significant changes in the microstructure of the liver. The integrity and morphology of the liver lobules were generally preserved. The hepatic vessels and sinusoidal capillaries showed a mild degree of dilatation and congestion. At higher magnifications, aggregates of small fat droplets were visible in the cytoplasm of hepatocytes (Figure 12).

The most significant histological changes in liver tissue were found in rats from the HFHCHD group. Hepatocytes had diffuse fatty degeneration, especially in the subcapsular and peripheral areas of the liver parenchyma. Significantly enlarged fat droplets and confluent lipid vacuoles occupied the entire cytoplasm of the liver cells. Against this background, the nuclei appeared compressed and pyknotic. The radial arrangement of the liver plates was completely disrupted (Figure 12).

**Figure 13:**
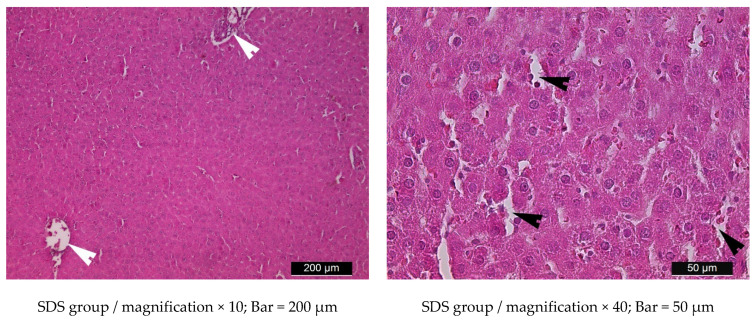

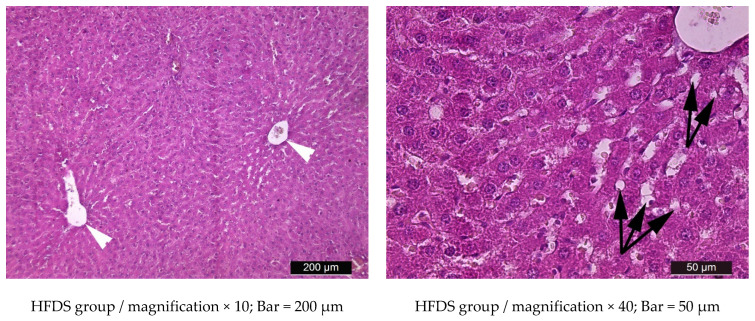
Light microscopic images of liver parenchyma in a rat from group SDS and a rat from group HFDS. White arrowheads indicate dilated central veins; Black arrowheads—dilated sinusoid capillaries; Black arrows—accumulation of small, single lipid vacuoles in the cytoplasm of hepatocytes. Hematoxylin–eosin staining.

In group SDS, the structure of the liver lobes was preserved. Slight dilatation of the central veins and sinusoidal capillaries was visible (Figure 13).

The simultaneous administration of steviol glycosides and a high-fat diet (group HFDS) had a positive effect on the micromorphology of the liver, as compared to the HFD group. Hepatocytes containing lipid vacuoles in the cytoplasm were found much more rarely, mainly in the periportal areas. Fat vacuoles in the cells were smaller in size. The structure of the hepatic plates and their arrangement were preserved, with the exception of some dilated blood vessels and sinusoidal capillaries (Figure 13).

### 3.6. Perirenal Adipose Depot Parameters and Histology

**Figure 14:**
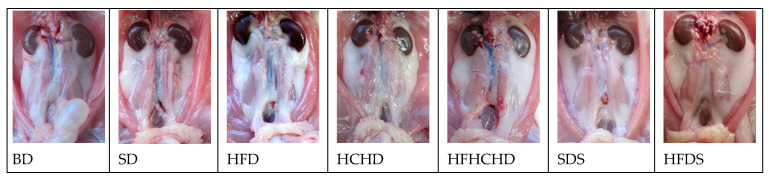
Ventral view of the perirenal fat depot in rats from all groups. The digestive system organs have been removed.

The perirenal fat depot was well developed and readily visible in rats from all groups. It was located between the ventral surface of *m. longissimus lumborum* and *m. psoas major* and the dorsal surface of the kidneys. Laterally, the depot extended to the soft abdominal wall. In animals from BD, SD, and SDS groups, adipose tissue next to the cranial poles of the kidneys was in insignificant quantity. In the caudal direction, the adipose tissue filled the angle between the above-mentioned muscles. In the abdominal cavity of rats from groups HFD, HCHD, HFHCHD, and HFDS, the perirenal adipose depot was hypertrophic, covering parts of *m. longissimus lumborum* and *m. psoas major* (Figure 14).

**Figure 15:**
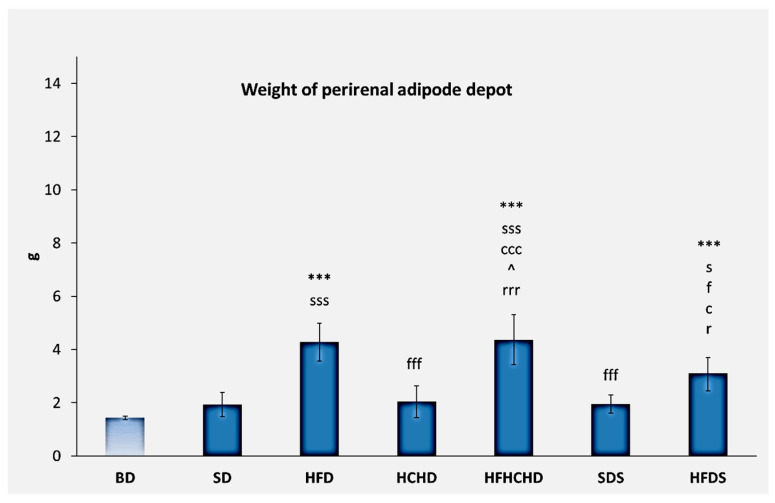
Perirenal adipose depot mass in rats (n = 6) before the start of the diets (BD) and in groups SD (n = 6), HFD (n = 6), HCHD (n = 6), HFHCHD (n = 6), SDS (n = 6), and HFDS (n = 6) after 5 weeks of application of the respective diets. Results are presented as mean values ± standard deviation. Statistically significant differences are indicated as follows: *** *p* < 0.001 vs. BD; s *p* < 0.05 vs. SD; sss *p* < 0.001 vs. SD; c *p* < 0.05 vs. HCHD; ccc *p* < 0.001 vs. HCHD; f *p* < 0.05 vs. HFD; fff *p* < 0.001 vs. HFD; r *p* < 0.05 vs. SDS; rrr *p* < 0.001 vs. SDS; ^ *p* < 0.05 vs. HFDS.

The baseline mass of the perirenal adipose depot was 1.43 ± 0.07 g. After applying the diets for a period of 5 weeks, the values increased significantly in HFD (4.28 ± 0.71 g) and HFHCHD (4.37 ± 0.94 g), as compared to BD, SD, HCHD, SDS (*p* < 0.001), and HFDS (*p* < 0.05). The values in HFDS also increased (3.10 ± 0.65 g) as compared to BD (*p* < 0.001) and SD, HCHD, and SDS (*p* < 0.05) (Figure 15).

**Figure 16:**
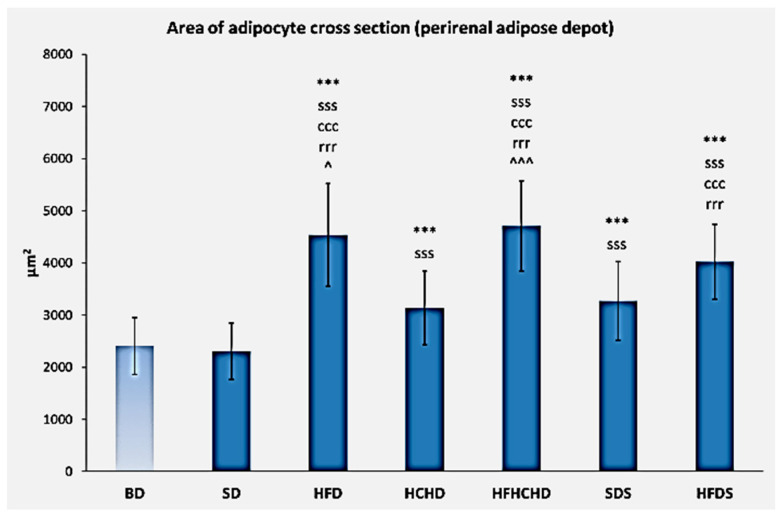
Cross-sectional area of adipocytes from the perirenal fat depot in rats (n = 6) before the start of the diets (BD) and in groups SD (n = 6), HFD (n = 6), HCHD (n = 6), HFHCHD (n = 6), SDS (n = 6), and HFDS (n = 6) after 5 weeks of application of the respective diets. Results are presented as mean values ± standard deviation. Statistically significant differences are indicated as follows: *** *p* < 0.001 compared to baseline (BD); sss *p* < 0.001 compared to SD group; ccc *p* < 0.001 compared to HCHD group; rrr *p* < 0.001 compared to SDS group; ^ *p* < 0.05, ^^^ *p* < 0.001 compared to HFDS group.

**Figure 17:**
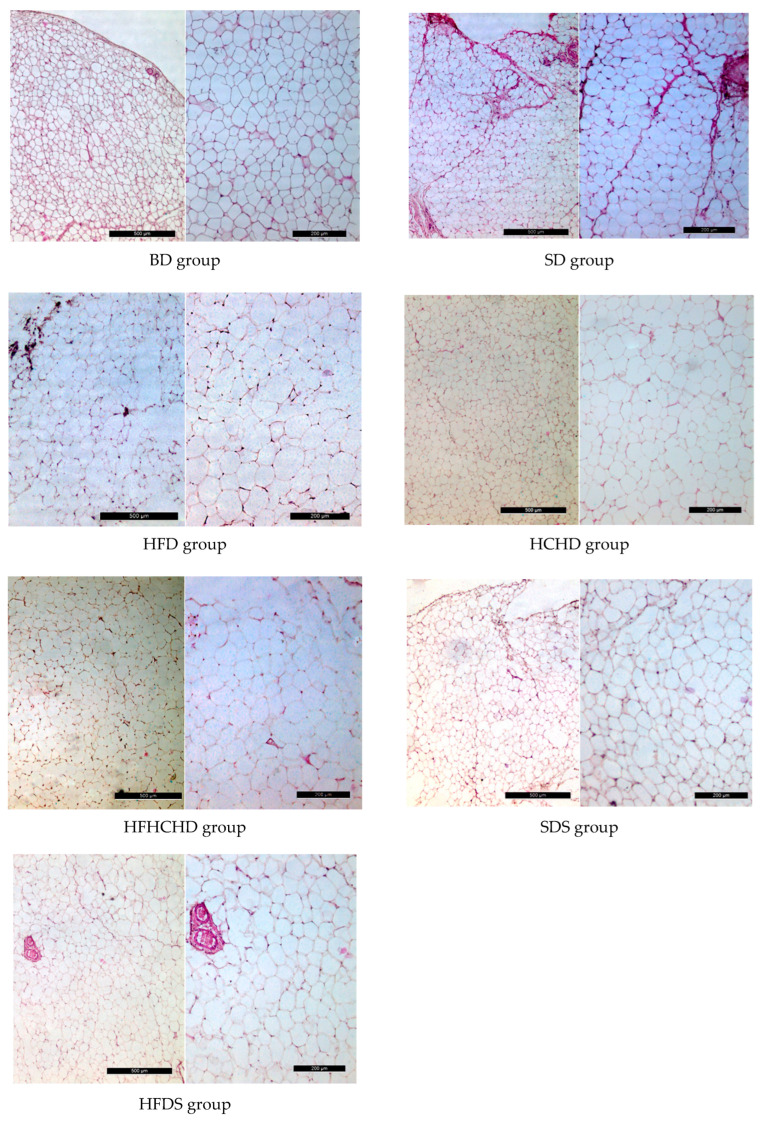
Light microscopic images of the perirenal fat depot in rats before the start of the diets (BD) and in SD, HFD, HCHD, HFHCHD, SDS, and HFDS groups. Staining with hematoxylin–eosin. Magnification X 5—Bar = 500 μm and magnification X 10—Bar = 200 μm.

The baseline values of the cross-sectional area of adipocytes from the perirenal fat depot were 2409 ± 548 μm^2^. After 5 weeks of application of the respective diets, the cross-sectional area of adipocytes increased statistically significantly in groups HFD, HCHD, HFHCHD, SDS and HFDS, as compared to baseline levels and to SD group (*p* < 0.001). A significant increase was also registered in groups HFD, HFHCHD and HFDS compared to HCHD and SDS groups (*p* < 0.001). In HFD and HFHCHD groups values were also higher, as compared to HFDS group (*p* < 0.05 and *p* < 0.001, respectively), (Figure 16 and Figure 17).

## 4. Discussion

Lipid profile indicators are widely used diagnostic markers in a number of diseases related to metabolic disorders. In addition to the metabolic dysregulation that occurs in the conditions of our experiment, the immediate short-term effect of the diets on lipid profile indicators should also be taken into account. Lard, used as the main additive in experimental high-fat diets, has high levels of monounsaturated fatty acids saturated fatty acids and lower cholesterol concentrations. These nutrients are the main factors influencing concentrations of different lipoproteins—indicators that are related and provide basic guidelines in the development of obesity-related diseases. It should also be taken into account that the concentration of total cholesterol is a function of the concentrations of different lipoproteins, and total cholesterol is a parameter that only gives a general idea of the metabolic disorders that have occurred. In addition, the Wistar rats used in our study are suitable for the purposes of our experiment, as they are susceptible to experimental diet-induced obesity and easily develop dyslipidemia [22]. After applying the experimental diets for a period of five weeks, a statistically significant increase in total cholesterol was recorded in the HFD and HFDS groups. The increases recorded in these groups are consistent with the changes in some of the other functional parameters, as well as with the histopathological changes in liver and visceral adipose tissue, which are discussed later in the section. Our results are in line with the results reported by other researchers—rats fed a high-calorie diet synthesize and accumulate more adipose tissue compared to rats on a standard diet, and increased visceral fat deposition is in most cases associated with dyslipidemia [23,24]. Obviously, lard included in the diets fuels the cholesterol-producing metabolic pathways. While patients with dyslipidemia will usually have low HDL-cholesterol, surprisingly, in our study, rats from the HFD and HFDS groups had a significant increase in HDLs. High-density lipoproteins (HDL) are one of the five main groups of lipoproteins, and their function and structural features determine their important role in lipid metabolism. HDL consists of cholesterol, triglycerides, and various apolipoproteins [25]. Apo A-I has anti-atherogenic activity. It is a cofactor for the enzyme lecithin-cholesterol acyltransferase (LCAT), which is involved in the transfer of excess cellular cholesterol from peripheral tissues to the liver, from where excess cholesterol can be excreted from the body [26]. This is the main protective role of HDL cholesterol—removal of excess cholesterol, improvement of endothelial function, and pleiotropic antioxidant and anti-inflammatory effects [27]. In the context of everything said so far, we can conclude that the increased levels of HDL-cholesterol recorded in our study probably represent a compensatory mechanism triggered by the intake of a larger amount of fat, which also changes the lipid profile. Nevertheless, it should be considered that short-term intake of high-fat diets may lead to a change in the biochemical composition of HDLs—the content of serum amyloid A and lipid hydroperoxides in HDL may increase, which is a major characteristic of lipoproteins with impaired function. Such change in HDL structure has been documented in both experimental animals and humans [28]. In conclusion, in certain pathological conditions, HDL can lose its beneficial anti-atherosclerotic properties and become dysfunctional, despite its increased levels. In relation to this statement, the significant increase in HDL recorded in the HFDS group may be an indication that Stevia extract does not improve the lipid profile, as reported in previous studies [29].

Important to analyze in the context of our study are also the changes in the concentrations of very low-density lipoproteins (VLDL) and triglycerides (TG). Triglycerides are the main component of adipose tissue and the main endogenous lipids synthesized by the liver. Triglycerides make up about sixty percent of the lipid content of VLDL, so it is not surprising that the recorded changes in the two parameters are identical. Numerous studies have demonstrated the relation between diets high in fats and carbohydrates and the occurrence of metabolic disorders such as obesity, insulin resistance, hypertriglyceridemia, histological changes in adipose tissue, and hepatic steatosis [30,31,32]. In our experimental study, an increase in triglyceride concentrations and VLDLs was recorded in the HFD, HCHD, HFHCHD, and HFDS groups. The highest and, respectively, statistically significant increases were in the HFHCHD group. Obviously, the combined administration of sucrose and lard provides the highest concentrations of metabolites needed for de novo synthesis of triglycerides in the liver, which subsequently has the most pronounced effect on triglycerides and VLDLs levels, and as discussed later, this diet had the most severe effect on liver tissue itself. These results confirm that the type of energy nutrients and the ratio between different nutrients can affect body weight gain, visceral fat, and the metabolism of very low-density lipoproteins, as well as other lipoprotein groups [33,34].

Liver function tests usually include alanine transaminase (ALT) and aspartate transaminase (AST), and these tests can determine the functional status of the liver, as well as the degree of damage, if any. Elevations in ALT and AST, when disproportionate to elevations in alkaline phosphatase and bilirubin, indicate hepatocellular disease [35]. AST and ALT values correlate with obesity but with a normal reference range, with values being higher in individuals with a higher body mass index. AST and ALT are also usually elevated in non-alcoholic fatty liver disease, in which dyslipidemia is present and other tests remain unchanged [36]. In the conditions of our study, after applying the experimental diets for a period of 5 weeks, no statistically significant changes in AST values were found, both compared to the baseline level and between the individual groups. Only changes in ALT and AST/ALT ratio were registered in the HFD and HFDS groups, which correlated with the established histopathological changes in the liver tissue and changes in some other enzyme parameters. Histopathological examination of the liver showed accumulation of lipid droplets. The increased ALT values in the HFDS group do not confirm the claims made by some researchers that steviol glycosides have a normalizing effect on lipid metabolism [37]. It seems that the high fat content in the HFD alone is sufficient to cause the observed changes. Moreover, the sweet taste of steviol glycosides present in HFDS may stimulate the appetite and food intake in rats from this group. In addition, the difference in the changes in the two enzymes comes from the fact that ALT is more specific for demonstrating liver dysfunction, and the changes in its concentrations are more prolonged than those of AST [38], which may explain results from our study. Moreover, normal concentrations of liver enzymes may vary between the different animal species due to genetically determined differences in the metabolic pathways, leading to interspecies variations in enzyme half-life. Thus, diagnostic sensitivity and specificity of liver enzymes may also vary between the different animal species [39,40].

The enzyme alkaline phosphatase (ALP) plays an important role as a dephosphorylating compound and is involved in liver metabolism. It is found in the cytosol of hepatocytes and in some other tissues such as bones, kidneys, and the mucosa of the small intestine. Elevated ALP values are used as a marker of impaired liver function or changes in the bone tissue [41]. In veterinary practice, elevated ALP levels are a common finding in routine biochemical tests performed when liver diseases are suspected. Nevertheless, it has to be mentioned that this biochemical indicator has a very high sensitivity but at the same time has a low specificity, as it is found in many tissues, and without additional tests, its diagnostic value remains controversial [42]. The histopathological changes registered in our study show accumulation of fat in hepatocytes, which leads to disruption of their structure. Oxidative damage is also suspected to affect hepatocytes in rats fed high-fat diets. Similarly to our study, other authors have also demonstrated the effect of feeding rats high-fat diets—increased lipotoxicity and lipid deposition in the liver have been reported, which also leads to changes in enzyme concentrations in the plasma [43]. In conclusion, elevated ALT and ALP can be considered the earliest biomarkers of liver dysfunction.

Bilirubin is a waste product of hemoglobin catabolism, which is included in the composition of bile, and subsequently it is eliminated through the feces. Thus, its concentration in the blood does not exceed normal values unless some pathological process is observed, especially one affecting the liver [44]. In the context of the results discussed so far, including histopathological changes, it is logical to assume that the increased bilirubin levels in the rats from the HFD group are a consequence of damage to the liver parenchyma. This, of course, should not exclude other hypotheses, especially considering that the most severe histological changes were found in the liver parenchyma of rats from the HFHCHD group. Recent studies have established the antioxidant activity of bilirubin and its action as a hormone that activates the nuclear receptor peroxisome proliferator-activated receptor α (PPARα) [45]. PPARα is expressed in hepatocytes, enterocytes, and vascular endothelium, and new studies have shown that unconjugated bilirubin acts as a novel endocrine ligand activating these receptors in mice fed a high-fat diet. The observed effects are associated with normalization of glucose and insulin levels [46]. Elevated plasma bilirubin levels are also associated with its positive antioxidant effects against increased reactive oxygen species (ROS) due to obesity, type 2 diabetes, and cardiovascular diseases [47]. Elevated bilirubin levels maintain oxidative balance [48]. In addition, according to some studies, bilirubin can inhibit lipid accumulation, prevent the development of steatosis, and regulate the overall energy homeostasis of the body by binding to PPARα [49]. Thus, elevated bilirubin levels in response to increased dietary intake of fats may represent a protective mechanism. Of course, such a statement is highly speculative in the context of our study, as the evidence is very limited. In conclusion, protective elevation of bilirubin and disorders of bilirubin metabolism may coexist.

One of the presumed mechanisms for the occurrence of some metabolic disorders is related to the development of chronic low-grade inflammation, caused by pro-inflammatory factors secreted by adipose tissue [50]. In this regard, our study examined the levels of nitric oxide (NO) in serum. Under normal physiological conditions, nitric oxide has an anti-inflammatory effect, but when nitric oxide is overproduced, it is considered an active pro-inflammatory mediator that provokes the development of inflammatory processes [51]. Nitric oxide is a significant component that contributes to the development of oxidative stress in animals fed a high-fat diet. Some researchers have reported that plasma and liver NO concentrations are significantly increased in rats fed a high-fat and high-carbohydrate diet. Moreover, some experimental studies have found that NO levels in liver tissue are higher in mice fed a high-carbohydrate diet compared to mice fed a high-fat diet. Higher levels of nitric oxide in the liver tissue stimulate the inflammatory response and lead to a more severe course of non-alcoholic fatty liver disease [43,52]. In line with these results, our study found the highest levels of nitric oxide in the HFHCHD group, where the most serious histopathological changes in the liver tissue were also recorded. Surprisingly, levels of NO in the SD group were higher as compared to the BD group, although the two groups were fed the same diet. This contradictory change could be age-related and can be attributed to the physiological growth of animals, as most strains of experimental rats have a significant growth rate until fourteen weeks of age [53,54]. The significantly lower values of nitric oxide in the groups with Stevia extract added to the diet (SDS and HFDS) confirm the anti-inflammatory and antioxidant properties attributed to steviol glycosides. The mechanisms by which steviol glycosides inhibit inflammatory reactions and oxidative stress are currently the focus of scientific research. Downregulation of receptor activities and signaling pathways and decreased degradation rates of antioxidants are among the suspected mechanisms [55,56,57].

The influence of high-carbohydrate and high-fat diets is also manifested morphologically. The presumed mechanisms are related to the uptake of fatty acids by adipocytes, which ultimately store them in the form of triglycerides. An excess of fatty acids inevitably leads to obesity. In parallel, hepatocytes absorb and accumulate fatty acids in the form of fat droplets, which leads to non-alcoholic steatosis of the liver. Excess fatty acids also induce increased hepatic lipogenesis, including cholesterol formation. Subsequently, lipids produced by the liver are transported and stored in fat tissue depots. High-carbohydrate diets containing monosaccharides and disaccharides rapidly increase the availability of substrates for lipogenesis in the liver [58,59,60]. Thus, the above-described pathophysiological mechanisms lead to lipid accumulation in liver tissue in the HFD, HCHD, HFHCHD, and HFDS groups. In the HFD group, the excess of lipids was clearly visible as lipid vacuoles in the cytoplasm of hepatocytes. Loading the rats from the HCHD group with sucrose caused the formation of aggregates of small fat droplets in the cytoplasm of hepatocytes, which proves the storage of excess carbohydrates as lipids through de novo lipogenesis [61]. The different morphological manifestations in the liver parenchyma in the HFD and HCHD groups support the results of Jensen [62], according to which dietary fat and cholesterol are the main drivers of the development and progression of non-alcoholic steatosis, while excess carbohydrates exert their effect mainly on the circulating lipid pool. Although the experimental period lasted only 5 weeks, the combined dietary regimen in the HFHCHD group resulted in diffuse fatty degeneration of hepatocytes. Rats from this group also had the highest values of the perirenal fat mass, size of adipocytes, VLDL cholesterol and triglyceride levels. In the HFDS group, histopathological changes in liver tissue were less severe. Obviously, replacing the sucrose component in the combined diet with steviol glycosides E 960/RA60 had a positive effect on the structure of the liver parenchyma compared to that in the HFHCD group. This positive effect can be attributed to the decreased caloric value of the diet.

Diets with high-fat content lead to visceral obesity. The perirenal fat depot demonstrated significant growth intensity. After 5 weeks of HFD, the perirenal depot increased its mass 2.8 times; in the HFHCHD group, the increase was 3.6 times, and in HFDS—3.5 times. At the same time, the increase in adipocyte size was not as significant as the increase in the mass of the perirenal fat depot. In none of the groups did the adipocyte size increase more than two times, which suggests both hypertrophy and hyperplasia are involved in the onset of dietary-induced visceral obesity. Generally, obesity is defined as excessive accumulation of fat tissue. In humans, the best method for diagnosing obesity is dual-energy X-ray absorptiometry (DEXA). Some studies have described the use of DEXA in laboratory animals [63]. However, this non-invasive technique may be difficult to apply in rodent experimental research. Because the fat depots in rodents are limited from the surrounding tissues by a well-developed fascia, it is possible to dissect them and separate them from the carcass in their integrity [64]. This made it possible to determine the mass of the perirenal fat depot in experimental rats. The perirenal adipose tissue in rats shows the highest responsiveness to dietary manipulations in comparison to the epididymal and subcutaneous fat depots [65]. In accordance with the above-mentioned features, the perirenal adipose depot in our study was selected as a basic morphological indicator, both at the macroscopic and microscopic levels, to evaluate the degree of obesity.

## 5. Conclusions

The high-fat, high-carbohydrate diet has the highest potential to induce hepatocellular injury and visceral obesity within a very short period of time, which are characterized by high levels of VLDL-cholesterol and triglycerides, high levels of serum NO, degeneration of hepatocytes, increase in the size of visceral adipocytes, and increased mass of the perirenal adipose tissue.

The high-fat diet induces less pronounced changes in the liver and visceral adipose tissue within a short period of time, which are characterized by increased levels of total cholesterol, HDL-cholesterol, ALT, ALP, total bilirubin, NO, presence of lipid vacuoles in hepatocytes, increased size of visceral adipocytes, and a moderate increase in the mass of the perirenal adipose tissue.

The high-carbohydrate diet produces insignificant changes in hepatocytes and visceral adipose tissue, at least in the short term.

Replacing sucrose in the HFHCHD with steviol glycosides does not improve lipid profile parameters and liver enzyme concentrations.

Replacing sucrose in the HFHCHD with steviol glycosides may alleviate hepatocellular changes and limit the accumulation of visceral fat to some extent and lower serum levels of NO. Whether this results from a decrease in the energy content of the diet or steviol glycosides possessing pharmacological effects has to be further investigated.

## 6. Limitations

The duration of the dietary regimens is very short because the study is focused on the onset of hepatocellular injury and visceral obesity. For this reason, results and conclusions may have limited clinical relevance.

The study does not analyze quantitatively the histological changes in liver tissue.

The study uses only serum levels of NO as an indirect inflammatory marker, while pro-inflammatory cytokines and oxidative stress markers have not been investigated.

## Data Availability

Dataset available on request from the authors.
